# Maladaptive Thinking Styles and Suicide Cognitions: Serial Mediation of Difficulties in Emotion Regulation and Loneliness

**DOI:** 10.1007/s11126-025-10164-4

**Published:** 2025-06-02

**Authors:** Ömer Faruk Akbulut, Sinan Okur, Muhammed Akat

**Affiliations:** 1 Quality Coordinatorship Unit, Afyonkarahisar Health Sciences University, Afyonkarahisar, Türkiye; 2 Department of Educational Sciences, National Defense University, Turkish Air Force Academy, Istanbul, Türkiye; 3 Faculty of Education, Department of Psychological Counseling, Karamanoglu Mehmetbey University, Karaman, Türkiye

**Keywords:** Maladaptive thinking styles, Suicide cognitions, Difficulties in emotion regulation, Loneliness, Serial mediation

## Abstract

There are many social, cultural, biological, and environmental factors that influence suicidal cognitions. Research aimed at understanding suicidal cognitions among university students may provide theoretical evidence for preventive studies on suicide. Accordingly, the present study aims to investigate how maladaptive thinking styles contribute to suicide cognitions through the serial mediating roles of difficulties in emotion regulation and loneliness. The sample consists of 617 university students (77.6% of the participants are female, *M*_age_ = 20.511, *SD* = 2.307). The correlation analysis revealed that maladaptive thinking styles were positively associated with difficulties in emotion regulation, loneliness, and suicide cognitions. Serial mediation analysis was conducted using the PROCESS macro (Model 6), which revealed that difficulties in emotion regulation and loneliness have a serial mediating role in the relationship between maladaptive thinking styles and suicide cognitions. The results have practical implications for developing intervention strategies targeting emotion regulation and loneliness to reduce suicide risk among university students. The research results are discussed and interpreted in the light of the literature.

## Introduction

Suicide is a serious public health problem among youth. According to the World Health Organization [80], suicide is the second leading cause of death among young people aged 15 to 29. Although suicide rates are high in some countries, rates are relatively low in countries such as Türkiye [3]. However, suicide rates in Türkiye have also started to increase in recent years [39]. According to the Turkish Statistical Institute [72], the crude suicide rate in Türkiye has reached its highest values ​​in the last three years. Furthermore, the same report revealed that young people accounted for 38.5% of all suicides. This increase in suicide rates is a concern for public health. Therefore, examining the risk factors for suicide and the mechanisms underlying suicidal behavior among university students is important for the development of preventive factors.

There are various approaches to understanding suicide in the literature [36, 38]. These theoretical approaches place a strong emphasis on the cognitive processes that define an individual's beliefs, attitudes, expectations, and perceptions about themselves, others, and the world [10]. The fluid vulnerability theory of suicide [57] focuses on Beck's cognitive theory and cognitive issues during the suicide process. This theory argues that cognition in the belief system makes some individuals more prone to suicide. These cognitions are conceptualized as suicidal cognitions and are defined as hopelessness beliefs about the self, others, and the future [10]. Suicidal cognitions consist of the components of unlovability, unbearability, and hopelessness [58]. Unlovability includes the individual's beliefs of inadequacy and worthlessness,unbearability includes the inability to cope with stress and difficulties in life; and hopelessness includes the belief that life problems are unsolvable [23]. In this respect, suicidal cognitions are a risk factor for suicidal tendencies and attempts [4]. It is crucial to unravel the mechanism of the suicide cognition system in order to provide preventive and therapeutic mental health services for individuals at risk of suicide [9]. This study endeavors to elucidate the mechanisms underlying suicide cognitions by examining both direct and mediate pathways.

### Maladaptive Thinking Styles and Suicide Cognitions

Maladaptive thinking styles are thoughts that a person uses to overcome difficulties and that adversely influence their emotions and behaviors [61]. These thoughts often have an inflexible structure. This way of thinking, which disrupts the functionality of individuals in their lives, is addressed in the literature with different concepts such as maladaptive thinking styles [60], irrational beliefs [22], cognitive distortions [5], early maladaptive schemas [83], and rumination [68]. Despite these different names, the common characteristics of these thinking styles are inflexible and irrational beliefs about the self, the future, and others. The concept of maladaptive thinking styles used in this study was examined through three core components within the cognitive analytic therapy framework: dilemmas, traps, and obstacles [60]. The dilemma component refers to thinking patterns in which the individual perceives only two rigid and extreme options in a situation (e.g., “*I must be perfect or I am a failure*”). The trap component involves negative inferences in interpersonal relationships (e.g., “*If I get close to others, they will eventually hurt me*”). The obstacle component reflects beliefs that desired goals cannot be achieved (e.g., “*No matter how hard I try, I will never succeed*”). These maladaptive thinking styles lead individuals to interpret experiences in a persistently negative and self-defeating manner [23].

Individuals with these ways of thinking assume that their negative thoughts will come true even if they engage in certain behaviors in their lives. These individuals have the belief that their actions will not change the negative outcome or will make it worse [30]. As a result, they experience hopelessness for both the present and the future [11]. Research emphasizes that suicidal cognitions involve thought patterns that one's life is unsatisfactory and not worth living [26]. These thoughts can range from a passive wish to die to active thoughts involving plans to end one's life [71]. In this respect, maladaptive thinking styles might increase the risk of suicide. Indeed, maladaptive thoughts are known to be a risk factor for suicidal cognitions and attempts (e.g., [25, 34]).

Theoretical approaches to understanding suicidal behavior provide insight into the impact of the cognitive system on suicide. The schematic suicide appraisal model [36], fluid vulnerability theory [59], the interpersonal-psychological theory [38], and the integrated motivational-volitional model [51] emphasize that a person’s negative appraisal of events leads to suicidal ideation. In fact, Rudd [57] states that individuals who have extremely negative beliefs about themselves and their environment are more likely to experience intense stress and anxiety, and as a result, their risk of suicide increases. Research has mostly focused on the relationship between maladaptive thinking styles and suicide attempts, the behavioral dimension of suicide (e.g., [17, 34]). However, although there are strong theoretical approaches to the relationship between maladaptive thinking styles and suicidal cognitions, the number of empirical studies in the literature is limited [24, 81]. Furthermore, the relationship between these two variables may also be influenced by other factors. Therefore, a contribution to the literature can be made by investigating both direct and indirect pathways of difficulties in emotion regulation and loneliness variables in the relationship between maladaptive thinking styles and suicidal cognitions.

### Difficulties in Emotion Regulation as a Mediator

The capacity to recognize and regulate emotions is important for one's mental wellbeing [14]. Cognitive approaches conceptualize emotions as a symptom of cognition and beliefs [43]. In particular, an irrational and unrealistic way of thinking about events can reveal negative emotions [22]. Therefore, as a result of maladaptive thinking styles, the frequency of experiencing negative emotions such as stress, anxiety, and hopelessness increases [48]. This situation makes it difficult for a person to regulate the intense emotions they feel [54]. Difficulty in emotion regulation is defined as an individual's lack of ability to cope with emotional situations or the use of maladaptive methods [29]. Although maladaptive thoughts might increase difficulties in emotional regulation, overwhelming and hard-to-manage emotions might, in turn, reinforce and perpetuate these maladaptive cognitions. This situation creates a vicious circle and negatively affects mental health [7]. Meta-analysis studies suggest difficulties in emotion regulation across many types of psychopathology, including depression [75], substance use [77], post-traumatic stress disorder [64], and suicide [56]. Failure to manage negative and intense emotions that arise as a result of maladaptive thinking styles can strengthen beliefs of hopelessness [62]. Individuals who have difficulty regulating their emotions often believe that there is no solution to their difficulties [82]. This situation leads to the development of suicidal cognitions. Emotional Cascade Theory [63] and Experiential Avoidance Model [13] consider suicidal behavior as a strategy of distracting attention from negative and intense emotional states and emotional avoidance. Some researchers have found that individuals with suicidal thoughts have difficulties in identifying, understanding, and expressing their emotions and that emotion regulation problems are the most important risk factors (e.g., [2, 27]). These theoretical frameworks provide support for the hypothesized mediating role of emotion regulation difficulties in the pathway from maladaptive thinking styles to suicide cognitions proposed in the present study. In the light of these explanations, it can be said that emotion regulation difficulties play a mediating role in the relationship between maladaptive thinking styles and suicidal cognitions.

### Loneliness as a Mediator

Loneliness is another concept that may play a mediating role between these variables. Maladaptive thinking styles adversely impact individuals’ social relationships. Individuals with maladaptive thinking styles often evaluate other people negatively [45]. This situation leads to their isolation and loneliness. Research indicates that maladaptive thinking styles are an important predictor of loneliness [42, 67]. Moreover, increased loneliness negatively influences a person's mental wellbeing [47]. Studies demonstrate that loneliness has a significant relationship with many types of psychopathology, especially depression and anxiety disorders [1, 78]. This situation poses a risk in terms of suicidal cognitions. Indeed, the Interpersonal Suicide Theory, one of the approaches explaining suicide, suggests that individuals develop hopelessness and a desire to die when they cannot change the structures of"thwarted belonging"and"perceived burden"in their lives [15]. The blocked belonging structure, among these structures, represents a situation that arises from the inability to meet the need for social interaction in a person's life [74]. The individual's experience of loneliness and social withdrawal leads to the emergence of thwarted belonging [37]. As a result of this situation, hopelessness and the risk of suicide increase [70]. According to some studies have shown that loneliness is one of the difficult emotions that can sometimes be unbearable and can lead to suicidal thoughts (e.g., [28, 66, 76]). These theoretical and empirical insights support the mediating role of loneliness in the relationship between maladaptive thinking styles and suicide cognitions proposed in the present study.

### Difficulties in Emotion Regulation and Loneliness as Serial Mediators

Based on the literature, previous research indicates that maladaptive thinking styles and suicide cognitions are associated with difficulties in emotional regulation and loneliness. However, these studies investigated difficulties in emotion regulation and loneliness separately in their relationship with maladaptive thinking styles and suicide cognitions. Recent studies indicate that difficulties in emotion regulation lead to loneliness in individuals [21, 40]. The findings of these studies revealed a direct positive relationship with loneliness. The findings indicate that the inability to control intense emotions contributes to feelings of loneliness. According to the Action Control Theory [8], individuals with difficulties in emotion regulation are situation-focused and are more likely to experience negative effects such as loneliness [65]. The existing literature supports the notion that there is a significant relationship between difficulties in emotion regulation and loneliness. The literature review in the introduction has identified a relationship between maladaptive thinking styles and difficulties in emotion regulation, as well as a relationship between loneliness and suicide cognitions. Given these findings, difficulties in emotion regulation and loneliness might serially mediate the link between maladaptive thinking styles and suicide cognitions.

### The Present Study

Suicidal cognitions are influenced by many social, cultural, biological, and environmental factors [46]. Although suicidal cognitions alone do not fully explain the complexity of suicide, examining the mechanisms underlying these cognitions is important to identify individuals who are highly susceptible to experiencing suicidal behaviors [23]. Additionally, as is known, the risk of suicide is common among young people [3]. In this context, research to understand suicidal cognitions among university students might provide theoretical evidence for preventive studies on suicide. The literature has frequently examined different predictors of suicidal cognitions [55, 73]. The role of maladaptive thinking styles, difficulties in emotion regulation, and loneliness in explaining suicidal cognitions has not yet been addressed. In this context, this study aimed to test the serial mediating role of difficulties in emotion regulation and loneliness in the relationship between maladaptive thinking styles and suicidal cognitions among university students. For this purpose, this study seeks to answer the following three hypotheses:

- *H1.* Difficulties in emotion regulation mediate the relationship between maladaptive thinking styles and suicide cognitions.
- *H2.* Loneliness mediate the relationship between maladaptive thinking styles and suicide cognitions.
- *H3.* Difficulties in emotion regulation and loneliness serially mediate the relationship between maladaptive thinking styles and suicide cognitions.

## Method

### Participants and Procedure

The sampling method for this cross-sectional study is convenience sampling. 617 students studying at different universities in Türkiye participated in the research. Among university students, 479 were female (77.6%) and 138 were male (22.4%) participants. The mean age of these individuals, who ranged from 18 to 43 years old, was 20.511 years (*SD* = 2.307). The majority of university students were pursuing undergraduate education at the time of data collection (*n* = 317, 51.4%). Most of the participants declared a medium socioeconomic position (*n* = 550, 89.1%).

The National Defense University Social and Humanities Research Ethics Committee has approved the study protocol (Reference Number = E-35592990-050.04-4015594). The research was conducted in compliance with the ethical standards established in the 1964 Declaration of Helsinki and its subsequent updates. The data of the research were collected through an online form, adhering to the principle of volunteerism. The design of this form allows participants to drop out of the study halfway through and can only be used if all questions are completed. Before beginning the survey, participants were provided with detailed information about the purpose, procedures, and voluntary nature of the study. They were explicitly informed that they could withdraw from the study at any time without any negative consequences. The informed consent form was embedded on the first page of the online form, and only those who agreed to participate by clicking the consent button were able to proceed to the questions. During the data collection process, the web link of the prepared form was shared on social media accounts (Whatsapp, Twitter, Instagram, etc.). All university students were informed about the study before participating in it. All participants gave informed consent before the study. No incentives were given to any participants during the study.

### Measures

#### Maladaptive Thinking Styles Scale (MTSS)

The MTSS was developed by Koç and Arslan [41] to assess individuals’ maladaptive thinking styles. The scale is a 10-item scale (e.g., ‘*I will either try to control everything and become overwhelmed, or I will lose control and panic*’) that assesses maladaptive thinking styles. Items are rated on a five-point scale from 1 (*strongly disagree*) to 5 (*strongly agree*). Higher scores indicate a high level of maladaptive thinking styles that impair the individual's functionality. The scale is three-dimensional. These have been called “dilemmas,” “traps,” and “obstacles”. The Turkish version of the scale exhibits robust psychometric properties, including very good construct validity (x^2^/df = 1.69, RMSEA = 0.03, SRMR = 0.05, GFI = 0.97, CFI = 0.93, and AGFI = 0.95), and good reliability (α = 0.81). In this study, the reliability of the scale was acceptable (ω = 0.85, α = 0.85, λ6 = 0.85).

#### Difficulties in Emotion Regulation Scale-8 (DERS-8)

The DERS-8 was developed by Penner et al. [53] to assess the difficulties in emotion regulation. The Turkish adaptation of the scale was carried out by [20]. The scale is an eight-item scale (e.g., “*When I feel down/bad, I feel out of control”*) that assesses difficulties in emotion regulation. Items are rated on a five-point scale from 1 (*almost never*) to 5 (*almost always*). High scores on the scale mean that there is more difficulty in regulating emotions. The scale has four dimensions:"purpose","impulse","non-acceptance"and"strategy". The Turkish version of the scale exhibits robust psychometric properties, including very good construct validity (χ2/df = 3.05, NFI = 0.96, CFI = 0.97, TLI = 0.95, and RMSEA = 0.07), and good reliability (α = 0.84). In this study, the reliability of the scale was acceptable (ω = 0.89, α = 0.89, λ6 = 0.88).

#### RULS-6 Loneliness Scale (RULS-6-LS)

The RULS-6-LS was developed by Wongpakaran et al. [79] to individuals’ loneliness. The Turkish adaptation of the scale was carried out by [33]. The scale is a 6-item scale (e.g., “*How often do you feel lonely?”*) that assesses loneliness. Items are rated on a four-point scale from 1 (*never*) to 4 (*always*). High scores obtained from the scale mean that the participants'level of loneliness increases. The scale is uni-dimensional. The Turkish version of the scale exhibits robust psychometric properties, including very good construct validity (CFI = 0.86, TLI = 0.84, NFI = 0.85, IFI = 0.86, and RMSEA = 0.12), and good reliability (α = 0.84). In this study, the reliability of the scale was acceptable (ω = 0.72, α = 0.66, λ6 = 0.70).

#### Suicide Cognitions Scale-Revised (SCS-R)

The SCS-R was developed by Bryan et al. [12] to assess the suicidal thoughts and beliefs. The Turkish adaptation of the scale was carried out by [6]. The scale is a 16-item scale (e.g., “*Nothing can save me*”) that assesses suicidal cognitions. Items are rated on a five-point scale from 0 (*totally disagree*) to 4 (*totally agree*). The higher possible scores obtained indicate that individuals have more suicidal cognitions. The scale is uni-dimensional. The Turkish version of the scale exhibits robust psychometric properties, including very good construct validity (CFI = 0.86, TLI = 0.84, NFI = 0.85, IFI = 0.86, and RMSEA = 0.12), and good reliability (α = 0.94). In this study, the reliability of the scale was acceptable (ω = 0.94, α = 0.94, λ6 = 0.95).

### Statistical Analysis

In this study, preliminary analyses were first conducted. Within the scope of these preliminary analyses, the descriptive statistics of the participants were examined, and then the normality values ​​and reliability values ​​of the variables were calculated. Furthermore, the relationships between variables were examined through correlation analysis. Following the preliminary analyses, the hypothetical model was tested with the PROCESS v4.1 macro plugin [31],Model 6). In this hypothetical model, the independent variable is maladaptive thinking styles, the dependent variable is suicide cognitions, and the mediating variables are difficulties in emotion regulation and loneliness, respectively. Additionally, gender and age were included in the model as covariate variables. In the conditional process analysis, direct and indirect paths on the dependent variable were tested. Finally, a bootstrapping analysis with 5,000 resamples and a 95% confidence interval was conducted to assess the significance of both the direct and indirect effects associated with the mediating variables. The absence of a zero value between the lowest and highest confidence interval values ​​was taken as the criterion for the statistical significance of the mediator variables [32]. These calculations were performed using SPSS 25.0, PROCESS v4.1 macro plugin, JASP 0.18.3.0 statistical package programs.

## Results

### Preliminary Analyses

All results obtained within the scope of preliminary analyses are given in Table 1. As a result of the analysis, the skewness and kurtosis values ​​of the variables are between −1.96 and + 1.96, which seems that the variables comply with the normal distribution criteria [69]. The fact that the reliability values ​​in all variables are above the 0.70 threshold proves that the scales have sufficient reliability [50]. In correlation analyses, all variables are positively and moderately significant with each other (*r* = 0.396–0.551, *p* < 0.01).

**Table 1:** Descriptive statistics, reliabilities and correlations for the study variables

Variable	Descriptive statistics and reliabilities							Correlations			
	Mean	*SD*	Skewness	Kurtosis	ω	α	λ6	1	2	3	4
1. MTS	22.800	8.103	0.380	−0.553	0.859	0.857	0.855	–			
2. DER	19.028	7.648	0.543	−0.528	0.892	0.891	0.887	0.551^**^	–		
3. Loneliness	11.882	3.233	0.586	−0.004	0.722	0.667	0.702	0.520^**^	0.396^**^	–	
4. Suicide cognitions	24.987	9.787	1.348	1.930	0.944	0.943	0.952	0.538^**^	0.522^**^	0.400^**^	–

*MTS* Maladaptive thinking styles, *DER* Difficulties in emotion regulation, ^**^ *p* < 0.01

### Serial Mediation Analysis

**Fig. 1:**
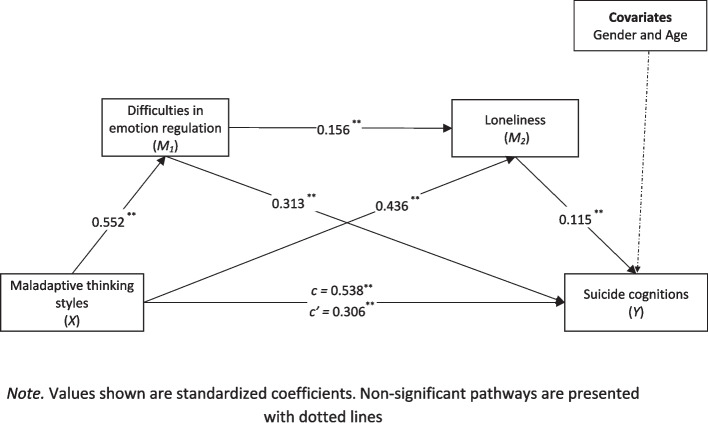
The result of serial mediation model, ^**^ p < 0.01. *Note.* Values shown are standardized coefficients. Non-significant pathways are presented with dotted lines

The findings of the serial mediation analysis conducted in line with the ultimate aim of the research are shown in Fig. 1. As seen in Fig. 1, maladaptive thinking styles positively predict suicide cognitions (*c* = 0.538, 95% CI = 0.570–0.731). When the mediator variables were included in the model simultaneously, the relationship between maladaptive thinking styles and suicide cognitions was still significant, but at a reduced level of significance (*c’* = 0.306, 95% CI = 0.271–0.469). This reveals that difficulties in emotion regulation and loneliness have a partial mediating role in this relationship (*c*—*c’* = 0.232, 95% CI = 0.173–0.295). In addition, maladaptive thinking styles positively predicted difficulties in emotion regulation (*a*_*1*_ = 0.552, 95% CI = 0.459–0.583) and loneliness (*a*_*2*_ = 0.436, 95% CI = 0.142–0.206). Furthermore, difficulties in emotion regulation (*b*_*1*_ = 0.313, 95% CI = 0.303–0.498) and loneliness (*b*_*2*_ = 0.115, 95% CI = 0.122–0.572) positively predicted suicide cognitions. Lastly, difficulties in emotion regulation positively predict loneliness (*d*_*21*_ = 0.156, 95% CI = 0.032–0.100).

The significance of the mediation findings obtained was tested using the bootstrapping method, and the results regarding the indirect effects obtained are presented in Table 2. Accordingly, the first indirect effect obtained was that the path from maladaptive thinking styles to suicide cognitions via difficulties in emotion regulation was significant (bootstrap = 0.173, 95% CI = 0.127–0.222). Another indirect effect finding, the path from maladaptive thinking styles to suicide cognitions, was significant via loneliness (bootstrap = 0.050, 95% CI = 0.010–0.091). The last indirect effect finding, the path from maladaptive thinking styles to suicide cognitions, was significant through difficulties in emotion regulation and loneliness, respectively (bootstrap = 0.001, 95% CI = 0.002–0.022).

**Table 2:** Standardized bootstrapping coefficients for the model

Path	Coefficient	95%CI	
		LL	UL
Indirect Effect			
MTS → DER → Suicide cognitions	0.173	0.127	0.222
MTS → Loneliness → Suicide cognitions	0.050	0.010	0.091
MTS → DER → Loneliness → Suicide cognitions	0.001	0.002	0.022

*MTS* Maladaptive thinking styles, *DER* Difficulties in emotion regulation, Based on 5000 bootstrap samples; *CI* Confidence interval, *LL* Lower limit, *UL* Upper limit

## Discussion

The present study investigated the serial mediating roles of difficulties in emotion regulation and loneliness in the relationship between maladaptive thinking styles and suicide cognitions. The study found that (1) difficulties in emotion regulation mediated the relationship between maladaptive thinking styles and suicide cognitions; (2) loneliness mediated the relationship between maladaptive thinking styles and suicide cognitions; and (3) difficulties in emotion regulation and loneliness, respectively, serially mediated the association between maladaptive thinking styles and suicidal cognitions. Consequently, all hypotheses are considered and discussed in detail below.

The findings answered the first hypothesis of this study (*H1*). Specifically, the study found that increased maladaptive thinking styles were associated with heightened difficulties in emotion regulation, which in turn predicted higher levels of suicide cognitions. The cognitive model emphasizes that negative emotions arise due to irrational cognitions about the stimulus rather than the stimulus itself [44]. Given the function of cognitions on negative emotions, maladaptive thinking styles may have an important impact on emotion regulation. Existing research indicates that maladaptive and irrational thinking significantly predicts difficulties in emotion regulation [19, 54]. Negative and uncontrollable intense emotions play an important role in suicidal behavior [52]. Difficulties in emotion regulation are a risk factor for suicidal cognition [62]. Difficulties in regulating emotions cause negative feelings to persist [75]. This situation influences suicidal cognitions by increasing beliefs of helplessness and hopelessness [82]. A meta-analysis study demonstrates that difficulties in emotion regulation are associated with both the cognitive and behavioral dimensions of suicide [16]. In addition to this research, there are many studies in the literature showing that there are strong relationships between emotion regulation difficulties and suicidal cognitions (e.g., [18, 27]). All these results align with the findings of this study. This finding demonstrates the potential importance of interventions targeting maladaptive cognitions and emotion regulation skills in suicide prevention efforts. The results highlight the need to address both cognitive and emotional mechanisms to better understand and reduce suicide risk.

People with maladaptive thinking styles tend to evaluate negatively [45]. As a result, they experience social isolation. Similarly, this study found that increased maladaptive thinking styles were associated with increased loneliness. Previous research indicates that maladaptive thinking styles are a significant predictor of loneliness (e.g., [35, 42, 67]). Furthermore, increased loneliness predicts high suicide cognitions. Previous studies have determined that increased loneliness and social isolation increase the risk of suicide [28]. The Interpersonal Suicide Theory emphasizes that the risk of hopelessness and suicide increases as a result of the failure to meet the need for social interaction in life [15]. This approach asserts that social isolation leads to the emergence of hindered belongingness and perceived burdensome components, which are significant risk factors for suicide [74]. Similarly, existing research reveals that loneliness is a significant predictor of suicidal cognitions [66, 70]. These findings answer the second hypothesis of the current study (*H2*). Interventions aimed at reducing maladaptive cognitions and alleviating loneliness may therefore be essential in mitigating suicide risk.

Lastly, the findings provided an answer to the third hypothesis of the study (*H3*). This study found that the serial mediating role of difficulties in emotion regulation and loneliness may also explain the association between maladaptive thinking styles and suicide cognitions. In this framework, higher levels of maladaptive thinking styles may lead to increased difficulties in emotion regulation [54]. Difficulties in emotion regulation, in turn, may increase loneliness [21]. This situation leads to increased loneliness, a negative emotion, and, as a result, increased suicidal cognitions [76]. While no previous research has directly examined the association between maladaptive thinking styles, difficulties in emotion regulation, loneliness, and suicide cognitions, there are studies focusing on the relationships among these variables individually. Various studies have reported that maladaptive thinking styles influence difficulties in emotion regulation [49] and loneliness [35], leading to changes in suicide cognitions [66]. Additionally, there is research demonstrating the impact of difficulties in emotion regulation on loneliness [40]. Therefore, the findings here indirectly, if not directly, support the conceptual view that there are serial mediators on the pathway from maladaptive thinking styles to suicidal cognitions. These results suggest that difficulties in emotion regulation and loneliness may operate in a serially manner, forming a critical pathway through which maladaptive thinking styles contribute to suicidal cognitions. This emphasizes the importance of addressing both emotional regulation capacities and loneliness simultaneously in preventive interventions.

### Limitations and Future Directions

There are several limitations to the research. First, as the study is cross-sectional, it is difficult to establish causal relationships between variables. Longitudinal and experimental studies may overcome this limitation. Secondly, the current study only examined the mediating roles of difficulties in emotion regulation and loneliness. Future studies can examine the mediating role of different cognitive, social, and emotional variables in the relationship between maladaptive thinking styles and suicide cognitions. In other words, the current model can be enriched by examining the mediation roles of other variables that may be potential mediators. Thirdly, the study data were collected from a non-clinical sample. Future studies should test this model using data from different samples. Another limitation of the study might be the potential biases arising from the self-report scales used in the study. Therefore, different measurement methods can be used. Finally, the participants consist of university students in Türkiye. This limitation should be considered when generalizing the research findings. Finally, testing the model with clinical samples could provide deeper insights into the mechanisms underlying suicidal cognitions in individuals at elevated risk, and help identify intervention targets that may differ from those in non-clinical populations.

### Implications

The current study showed that maladaptive thinking styles can link suicide cognitions through difficulties in emotion regulation and loneliness. These two mediating variables may be risk factors in the relationship between maladaptive thinking styles and suicidal cognitions. In this context, it can be recommended to implement psycho-educational programs based on cognitive behavioral therapy to recognize students'maladaptive and illogical cognitions and to replace these cognitions with healthy thought structures. These programs can also include studies designed to enhance students'social interactions and emotion regulation skills. For practitioners, integrating these approaches into individual and group counseling sessions may help reduce suicide risk among vulnerable students. In addition, higher education institutions should provide peer counseling services and organize social, cultural, and sporting activities to aid in the socialization of university students who are experiencing high levels of loneliness. Such activities can support students'social development and play a protective role in terms of suicide risk. For policymakers, supporting the implementation of campus-wide initiatives and mental health promotion strategies will be essential to create inclusive and supportive educational environments. Furthermore, for researchers, future studies should continue to explore the dynamic interplay between cognition, emotion regulation, and social factors, particularly via longitudinal and intervention-based designs to strengthen the evidence base for targeted suicide prevention programs.

### Conclusion

This study empirically supported previous theoretical explanations by establishing a relationship between maladaptive thinking styles and suicidal cognitions among college students. The literature contains a limited number of studies revealing this relationship. Notably, the serial mediating effect of difficulties in emotion regulation and loneliness could further elucidate the link between maladaptive thinking styles and suicide cognitions. The study emphasizes that maladaptive thinking styles can lead to challenges in emotion regulation, which can then trigger loneliness, ultimately influencing suicide cognitions. The findings of this study are important for planning mental health services designed to prevent suicide risk. Furthermore, this study contributes not only to the academic literature but also to mental health professionals by identifying the variables that mediate the relationship between maladaptive thinking styles and suicide cognitions.

## Data Availability

Data will be available on request.

## Declarations

### Pre-registration Statement

This study was not pre-registered.
